# Vienna Summer School on Oncology: how to teach clinical decision making in a multidisciplinary environment

**DOI:** 10.1186/s12909-017-0922-3

**Published:** 2017-06-06

**Authors:** Carola Lütgendorf-Caucig, Philipp A. Kaiser, Alexandra Machacek, Cora Waldstein, Richard Pötter, Henriette Löffler-Stastka

**Affiliations:** 1 Department of Radiation Oncology, Comprehensive Cancer Center, Christian Doppler Laboratory for Medical Radiation Research for Radiation Oncology; Medical University of Vienna/General Hospital of Vienna, Vienna, Austria; 2 International Students Office, Medical Students Association, Medical University Vienna, Vienna, Austria; 3 Department of Psychoanalysis and Psychotherapy, and Teaching Center, Medical University of Vienna/General Hospital of Vienna, Waehringer Guertel 18-20, A-1090 Vienna, Austria

**Keywords:** Summerschool, Cancer education, Clinical decision making

## Abstract

**Background:**

Clinical decision making in oncology is based on both inter- and multidisciplinary approach. Hence teaching future doctors involved in oncology or general health practice is crucial. The aim of the Vienna Summer School on Oncology (VSSO) as an international, integrated, undergraduate oncology course is to teach medical students interdisciplinary team communication and application of treatment concepts/algorithms in a multidisciplinary setting.

**Method:**

The teaching is based on an inter- and multidisciplinary faculty and a multimodal education approach to address different learning styles. The participants rated their satisfaction of the program voluntarily after finishing the course according to a grading scale from one (not good) to five (very good). The learning success was assessed by a compulsory pre-VSSO and post-VSSO single choice questionnaire.

**Results:**

Program organisation was rated with a mean score of 4.47 out of 5.0 (SD 0.51), composition of the program and range of topics with a mean score of 4.68 (SD 0.58) and all teachers with a mean score of 4.36 (SD 0.40) points. Student evaluation at the beginning and end of the program indicated significant knowledge acquisition –i.e., general aspects of cancer: median 8.75 points (IQR 7.5–9.4) vs.10.0 points (IQR 9.4–10.0) *p* = 0.005; specific aspects of cancer: median 4.87 points (IQR 3.33–5.71) vs. 8.72 points (IQR 6.78–9.49) *p* ≤ 0.001, respectively.

**Conclusion:**

Even though the participants represent a selection of students with special interest in cancer, the results of the VSSO indicate the benefit of an inter- and multidisciplinary teaching approach within an oncology module.

**Electronic supplementary material:**

The online version of this article (doi:10.1186/s12909-017-0922-3) contains supplementary material, which is available to authorized users.

## Background

Cancer is one of mankind’s severest diseases, causing a death rate of 25% in developed countries. Due to advances in cancer screening, diagnoses and treatment, future doctors will face an increasing number of cancer patients and survivors.

Consequently, an increased need of training and knowledge of active cancer care among doctors emerges [1]. The World Health Organisation (WHO) and the International Union Against Cancer (UICC) [2] gave the recommendation that medical students should spend at least a two weeks training in oncology. Although undergraduate training in oncology is regarded as useful for future doctors of all medical fields [3], there is no consensus in medical faculties worldwide about what should be taught at the undergraduate level [3–5]. Teaching modern cancer care must cover fundamental understanding of translational science and methods, epidemiology, diagnostics, basic knowledge in surgical, medical and radio-oncological cancer treatments, as well as communications skills and palliative care. A major challenge lies in developing core skills in oncology. In addition, clinical decision making in oncology is based on interdisciplinary team communication and application of treatment concepts/algorithms in a multidisciplinary setting. This requires a high level fundamental pre-clinical and clinical knowledge and the ability to apply the acquired knowledge.

Teaching about cancer in Europe is often fragmented [5–9] as there is currently no common syllabus among medical schools about how to teach the multi- and interdisciplinary approach of oncology, [9]. From this situation arose the need for an international, integrated, multidisciplinary oncology training for undergraduates, which has prompted the establishment of several summer schools on oncology [8, 10–14]. Nevertheless, the summer schools differ in their content, aims and student selection. The article will focus on the recent development and methodical approach of the “Vienna Summer School on Oncology” (VSSO; (www.meduniwien.ac.at/vsso/) as an international, integrated, undergraduate oncology course for medical students in the final phase of their studies. The VSSO was first held in 1999. Since 2001, the course’s location has alternated between Vienna and the collaborating WHO-Cancer Centre for Cancer Education in Groningen (WHOCCCE; www.isoms.nl) under the auspices of the International Union against Cancer (UICC) [10, 11, 14].

In 2015 the methodical concept of the VSSO was revised and renewed in terms of the course composition. We support the hypothesis that the integration of different teaching modalities supports the knowledge acquisition for clinical decision making in a multidisciplinary environment like oncology: We hypothesize, that case-based blended learning and critical judgement of evidence-based literature results in better exam scores.

From 2016 onwards, the summer schools in Groningen/The Netherlands and in Vienna/Austria are supported by the European Society of Radiotherapy and Oncology (ESTRO, for the ongoing programmes for undergraduate multidisciplinary teaching see under "School" in www.estro.org), European Society of Surgical Oncology (ESSO) and the European School of Oncology (ESO).

## Methods

### Course design

The aim of the VSSO is to familiarize students with general cancer care, to reduce their fear of patients with malignancies, and to teach them about cancer-related problems in other countries. Students learn about cancer research as well as new developments in diagnostic and treatment technology (invasive and non-invasive). Program candidates are undergraduate medical students interested in clinical oncology, basic research, and the cultivation of international contacts. Participation is limited to thirty students.

The organizing committee consists of radiation oncologists, medical and surgical oncologists and a diagnostic radiologist from the Medical University of Vienna (MUW) and the Comprehensive Cancer Center (CCC) in Vienna in collaboration with the WHO-CCCE in Groningen, Netherlands. Austrian students from the MUW are also recruited to help students with general organization and social programs. All courses are held at the Vienna General Hospital and MUW.

Moodle (an open-source software learning management system) is used for information exchange and learning evaluation. A major advantage of the Moodle platform is that many students are already familiar with it.

The program is comprised of two parts: clinical (T1) and research (T2).

T1: The goal of the clinical track is to familiarize students with cancer care in a hospital setting. Students accompany physicians through the ward and attend lectures on communicating with the terminally ill.

T2: The goal of the research track is to introduce the various aspects of translational oncological research. Participants are introduced to different facilities and laboratories and attend lectures by experienced researchers from different backgrounds.

### Concept and methodological approach of track T1 (eLearning-blended learning)

Clinical reasoning is often regarded as consisting of intuitive and analytical components [15, 16]. Research on mental processes suggests that disease patterns are stored in “frames,” “clinical scenarios,” “semantic networks/qualifiers,” or “illness scripts.” Repeated presentation and analysis of clinical cases is known to be crucial to the learning process [16, 17]. Implementation of an interactive, case-based teaching method in the Curriculum of the MUW was initiated in 2014 and in the VSSO program in 2015. Evaluation of these curriculum-elements at the MUW has shown to have a positive effect on the learning process [18, 19]. The main effect is achieved through case-based exercises in clinical reasoning seminars and by learning the patient’s perspective through bedside teaching methods [20]. Case-based learning provides a solid basis for clinical reasoning processes in general [20, 21], additionally, the efficacy of training increased when adequate questions methods were added [21].

### Concept and methodological approach of T2 (eLearning)

The course covers the following competencies: (1) judging the relevance of the topic through evidence-based knowledge and clinical literature, (2) formulating targeted research questions, (3) explaining and arguing methodology. Some students may additionally develop a specific research design, apply and implement a project in order to address questions relevant to diagnostic procedures or treatment issues. Interaction with lecturers provides a research-stimulating environment in which professional mentors and professors function as role models for scientific thinking. Kahneman recommends “Thinking in fast and slow pathways” [22]. “Slow pathways thinking” is essential for analysing complex diseases such as cancer, as this illness is often loaded with diverse, affectively hard to contain meanings. Clinical reasoning is considered to consist of intuitive and analytical components [23] referring to two systems in the human mind. The fast affective information from mesolimbic pathways, as well as the frontal and the parietal cortex linked to the analytic system of decision-making need to be combined to produce our decisions and control the higher-order thinking and complex reasoning, also mediating current actions and future consequences [22].

### Course program (Fig. 1)

The educational program lasts for seven days. As the students come from different countries, the educational program consists of various modules – T1 especially to foster clinical reasoning and decision making [23] - and addresses different learning styles: (1) pre-module and presentations (2) classical lectures (3) workshops (4) blended learning (5) knowledge check.

1. As a pre-module all students are requested to send an abstract upfront with an oncologic topic related to cancer and cancer care in their home country/institution. Students in the clinical track (T1) had to prepare an abstract related to a clinical topic and students in the research based track (T2) an abstract dealing with a preclinical or translational research subject related to oncology. A faculty member at the home medical school should supervise the preparation of the abstract. T1 students present a poster on the topic of their abstract and the T2 students give a short oral presentation. All abstracts, posters and presentations are reviewed by faculty members of the Medical University Vienna. The aim of the abstracts and poster presentations during the VSSO is, besides learning about cancer care in different countries, to learn how to prepare an abstract, a poster and how to make a presentation at an international meeting.
2. Lectures provide an overview of general aspects of cancer starting with teaching declarative knowledge (e.g., pathophysiology), fostering associative learning by application of knowledge within specific problems (e.g., diagnostic algorithms) and training of procedural knowledge in certain cases (e.g., providing specified authentic care and treatment). The lectures are given by experienced teachers who encourage students to participate in discussions.
3. Workshops offer an insight into specific fields of oncology for each track. The students have the possibility to get together with small groups familiar with techniques and work flows related to their special interest, including also team-teaching as didactic method.
4. Blended learning is a validated emerging paradigm for science education with in-class problem solving and computer-mediated activities in small groups under the supervision of teachers [24]. Problem based learning for each track and virtual patient cases are integrated. Virtual cases [18] with case-based interactive questions [21] are analysed in small groups from the perspective of a diagnostic radiologist, a surgeon, a medical oncologist or a radiation oncologist of the teachers, face-to-face and computer mediated [19]. In the end treatment strategies are discussed with all groups and teachers during an oncology round.
5. Upfront the VSSO and after the last lecture a compulsory knowledge check (Additional file 1) in terms of a single choice test, is performed. For each knowledge-check a maximum of ten points can be achieved. The knowledge-check includes eight questions concerning general aspects and thirty-nine questions covering specific aspects of cancer. The questions are related to the key messages of the lectures. The upfront knowledge check helps the teachers to evaluate the students’ knowledge they bring to the VSSO, the one at the end reflects the knowledge the students gain. In addition, knowledge checks, performed right after lectures, support the information process and learning [25].

**Fig. 1:**
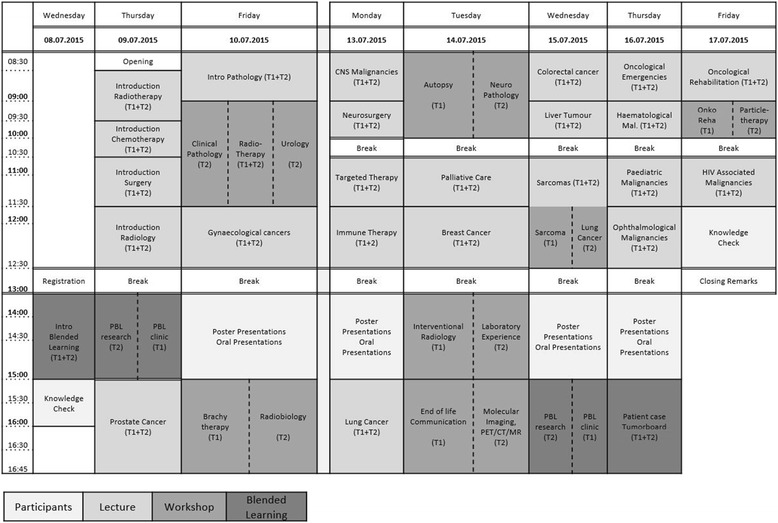
Vienna Summer School on Oncology 2015 Program. Legend: *light grey*…*Participants, grey*…*Lecture, dark grey*…*Workshop*, *dark-bold* *grey*…*Blended Learning*

### Evaluation

#### Program

Each participant is asked to answer voluntarily an anonymous electronic evaluation at the end of the program. The participants were asked to rate organisational matters like course program, organisation, range of topics and social program as well as educational matters like every lecture, workshop and teachers. They were asked to score every question according to a grading scale from one (not good) to five (very good). The students were also encouraged to give feedback using a “further comments” section.

#### Knowledge check

Before the VSSO and after the last lecture a compulsory knowledge check in terms of a single choice test was performed. The knowledge check (Additional file 1) included eight questions concerning general aspects and thirty-nine questions covering specific aspects of cancer. For each knowledge-check maximum of ten points could be achieved. The questions were related to the key messages of the lectures. The upfront knowledge check helped the teachers to evaluate the students’ knowledge they bring to the VSSO, the one at the end reflects the knowledge the students gained. In addition, knowledge checks - especially those including feedback and explanations for right or wrong answers [18, 19, 21] - performed right after lectures, support the information process and learning, as well as the analytical thinking [25] necessary for clinical reasoning and decision making as described by Kahneman [22]. Case-based question driven learning is effective, if the students get ad hoc answers to their right or wrong argument or clinical decision-making process [15]. An overview of the questions is given in the Appendix (Additi﻿onal file 1). To provide an interactive low stake test example, we asked within the presentation of a case “Which of the following factors is affecting the patient’s prognosis the most?” and gave the explanations of the possible answers (e.g., patient’s age, patient’s co-morbidities, biological behaviour of the tumour, the tumour stage, patient’s sex) automatically right after the student had answered the question in the eLearning program, but the answer could only be submitted once. As an example for a high stake question we asked “Next step for this patient with a high suspicion of prostate cancer and a negative transrectal biopsy is”: e.g., Androgen deprivation therapy, radical prostatectomy, re-biopsy, PSA control in 3 months). All the questions support patient-centred clinical decision making, and the knowledge that has necessarily to be retrieved therefore. In order to test multidisciplinary thinking and arguing as well as translational knowledge, questions like “ The diagnosis of cancer has to be usually confirmed by: e.g., radiation oncologist, surgeon, radiologist, medical oncologist, pathologist” (low stake) or “Which side effect is typical for 5-FU? cholinergic syndrome, skin rash, increased transaminases, hand and foot syndrome” (high stake) or further questions on the molecular or biochemical level (“Which chemotherapy is a typical alkylating agent?”), or, concerning the inclusion of different disciplines for instance for technical issues (What is the Bragg peak? E.g., the perfect time point to start an ion beam treatment, a boost to the residual treatment given by proton or carbon ion therapy, a small mountain in Austria, not far from MedAustron, the maximum of the characteristic energy loss curve of charged particles”).

#### Statistical analyses

The evaluation of the VSSO evaluation and knowledge checks is in accordance with the regularities of the data protection committee of the Medical University of Vienna. The results of the Moodle electronic course evaluation forms and electronic single choice test were extracted into excel tables. The completed data records were statistically evaluated with SPSS 22.0 (SPSS Inc., Chicago, IL) for Mac OS. For the results the mean values and the corresponding standard deviation (SD) were calculated. Concerning the results of the knowledge checks median and the interquartile range (IQR) was calculated. The results of the knowledge check at the beginning and the end of the course were compared using a t-test.

## Results

### Organisation and program (Table 1)

Each participant is asked to answer an anonymous electronic evaluation at the program’s conclusion. The participants were asked to rate the program’s organisation, range of topics, and social component. The students were also encouraged to provide general commentary about the program’s quality.

**Table 1:** Evaluation of the VSSO 2015

	*5 (excellent)*	*4*	*3*	*2*	*1 (poor)*	*Mean (STD)*
Composition of the program	13 (69%)	5 (26%)	1 (5%)	0 (0%)	0 (0%)	4.68 (0.58)
Organisation	9 (47%)	10 (53%)	0 (0%)	0 (0%)	0 (0%)	4.47 (0.51)
Range of topics	13 (69%)	5 (26%)	1 (5%)	0 (0%)	0 (0%)	4.68 (0.58)
Social program	7 (37%)	9 (47%)	2 (11%)	1 (5%)	0 (0%)	4.16 (0.83)

Legend: Rating range from 5 (excellent) to 1 (poor) on the lime survey given in total (and relative) frequency. The evaluation was on a voluntary basis; the students were asked to rate only the workshops they really attended; multiple ratings were not allowed

The students showed high level of satisfaction with the composition of the program and the range of topics by a mean score of 4.68 (SD 0.58) out of five.

Evaluation of the program’s teachers and methods indicates a similarly high overall satisfaction, with a mean of 4.36 (SD 0.40) points for all teachers. Workshop teachers were generally better evaluated than the lecturers. Targeted therapy, HIV-associated malignancies, and immunotherapy were rated as the three best lectures; autopsy, sarcoma, urology, and end-of-life communication were rated the best workshops.

Most student comments about the VSSO were very positive. No student indicated that the lectures and workshops failed to meet their learning expectations. The most notable complaints pertained to tight and early-morning scheduling. The students recommended maintaining the composition of the program, but extending its duration from 7 to 10 days.

### Knowledge checks (Fig. 2)

The knowledge check included eight questions concerning general aspects and thirty-nine questions covering specific aspects of cancer. Within each knowledge check a maximum of ten points could be achieved. The exam dealing with general cancer education has shown a median 8.75 points (IQR 7.5–9.4) before the course; at the program’s end a median of 10.0 points (IQR 9.4–10.0) was achieved. Evaluations of the exam covering specific aspects of cancer indicated even greater knowledge acquisition. At the starting evaluation a median of 4.87 points (IQR 3.33–5.71) was reached; at the program’s completion, a median of 8.72 points (IQR 6.78–9.49). The gained knowledge about general and specific aspects of cancer was significant, with a *p*-value of *p* = 0.005 and a *p*-value of *p* ≤ 0.001 respectively.

**Fig. 2:**
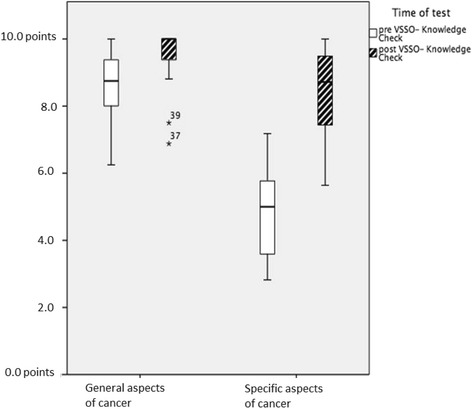
Timepoints of Knowledge Check. Legend: *white*…pre-VSSO knowledge check, *black*…post-VSSO knowledge check

## Discussion

Given that the incidence and prevalence of cancer is continuously increasing it is very important to arouse the future doctors’ interest into the inter- and multidisciplinary approach in oncology. There is clear evidence that cancer education is underrepresented in the medical curriculum in many countries [9]*.* One reason might be the complexity of the inter- and multi-disciplinarity in oncology. Medical students with a special interest in oncology are often dependent on a special postgraduate training, because the exposure during the undergraduate curriculum is very limited [3, 26]. But not only will the future oncologist have to deal with cancer patients during their professional career, but especially those doctors that are involved in general health practice. This points out how important undergraduate oncology teaching is, considering that we will face an increase in cancer patients.

Oncology is based on multi- and interdisciplinarity and makes teaching with the goal to support future physicians applying the gained knowledge very complex [27]. Our approach to provide adequate containment of the interdependencies between the specific fields in diagnosis and treatment as it was offered in the blended learning program led to acceptable examination performance, especially for the specific aspects of cancer. This exceeds previous findings [28] and can be discussed as a suggestion for improvement in clinical teaching. Additionally the research track and presentation of experimental and applied research projects and facilities in oncology assist the understanding of reciprocal effects on a translational pathognomonic and pathoplastic level. The integration of blended learning elements to prepare students for this research track (providing several different topics, free eligible time for acquisition of knowledge) leads to a satisfying learning environment, which is consistent with previous findings [19] and now can be extended to the field of oncology. The evaluation of the VSSO reflected a high acceptance from students who appreciated the course composition including the multidisciplinary approach. The workshops and blended-learning modules showed in general a better evaluation compared to the classical lectures. This supports the hypothesis, that students reduce their fear of interaction in small groups with enthusiastic tutors at a young age. One reason for the high acceptance might be due to the structured organisation of different teaching models supported by the Moodle platform, small group teaching and the face-to-face interaction. Within the framework of the course composition the participants achieved their knowledge through a combination of self-study, small group study and in-class study. The efficacy of the course is well documented by the results of the knowledge checks. The increase of knowledge about general and specific aspects of cancer as well as applying the knowledge regarding the clinical reasoning processes within the oncology round improved during the VSSO. These results indicate that when teaching complex interdisciplinary topics like oncology, addressing different learning styles and teaching approaches are leading to a great learning success.

A limitation of the excellent evaluation and results is that the participating students represent a selection of students with special interest in oncology and not all participants used the possibility to give feedback in terms of the electronic evaluation form.

While improvements are always possible the upcoming VSSO 2017 will have several changes in the program based on our experience and the remarks of the students. Concerning the schedule, the duration will be extended to ten days, as recommended by the WHO and UICC [13], while maintaining the range of topics*.* As the participants come from different faculties across the world, the pre-module will be expanded in order to adjust their level in advance. In addition to the abstract, which has to be sent in advance, the pre-VSSO knowledge check and one inter-active patient case will be part of the pre-module. Further, the pre-module will include one inter-active patient case, which has to be prepared and discussed in small-groups via Moodle and thereafter presented during the summer school under the guidance of a tutor. This flipped classroom strategy should also improve the interaction between students and teachers as well as the acceptance of the Moodle-based blended learning tools.

## Conclusions

In conclusion, students should practice everyday patient evaluation and learn the value of interdisciplinary team communication as well as the application of treatment concepts/algorithms and crucial disease pathophysiology. Clinical decision making should proceed based on the results of prototypic case-based derived knowledge supporting associative and procedural learning processes. Students should learn the value of inter- and multidisciplinary team approaches in oncology. Hence we support the implementation of a mandatory inter- and multidisciplinary ten-day module on oncology with didactically sound teaching methods for all undergraduate students to arouse the interest in oncology. Taking developments of precision medicine, changes in health care systems, emerging economically driven decisions and the connected ethical considerations into account, students should be prepared for an interdisciplinary work and communication. Multidisciplinary teaching approches e.g., [14] are therefore an emerging need in under- and postgraduate Cancer Education.

## Additional file

Additional file 1: The exam comprises two steps. First, declarative knowledge about general aspects of cancer is tested, including diagnostic and therapeutic procedures, going further to questions about preventive strategies and interdisciplinary cooperation. Second, associative learning is tested in questions about translational (research) aspects covering the competencies of collaboration and communication with adjoined disciplines, procedural knowledge concerning professionalism, scientific competences and competencies of taking the role of a manager, health advocate and scholar (Frank, 2005). (DOCX 38 kb)

## Abbreviations

CCC: Comprehensive cancer center
ESO: European school of oncology
ESSO: European society of surgical oncology
ESTRO: European society of radiotherapy and oncology
Fig: Figure
IQR: Interquartile range
MUW: Medical University of Vienna
SD: Standard deviation
T1: Clinical track
T2: Research track
Tab: Table
UICC: International Union Against Cancer
VSSO: Vienna Summer School on Oncology
WHO: World Health Organisation
