# Supplementary material for: Vienna Summer School on Oncology: how to teach clinical decision making in a multidisciplinary environment
Source: BMC Med Educ. 2017 Jun 6;17:100. doi: 10.1186/s12909-017-0922-3 (PMC5461756; doi:10.1186/s12909-017-0922-3)
Supplement: Additional file 1: — The exam comprises two steps. First, declarative knowledge about general aspects of cancer is tested, including diagnostic and therapeutic procedures, going further to questions about preventive strategies and interdisciplinary cooperation. Second, associative learning is tested in questions about translational (research) aspects covering the competencies of collaboration and communication with adjoined disciplines, procedural knowledge concerning professionalism, scientific competences and competencies of taking the role of a manager, health advocate and scholar (Frank, 2005). (DOCX 38 kb) [file 12909_2017_922_MOESM1_ESM.docx]

GENERAL ASPECTS OF CANCER

1. The goal of cancer screening is

- to detect the primary cancer site
- to diagnose a specific cancer at an early stage
- to prevent all kind of cancer
- to prevent cancer at a specific site

1. The concept "the earlier a cancer is diagnosed and the treatment starts, the better the patient´s prognosis" is:

- true for all kind of cancers
- true for most of kind of cancers
- true for only the minority of cancer
- tumour stage and time to treatment are not relevant

1. The diagnosis of cancer has to be usually confirmed by:

- radiation oncologist
- surgeon
- radiologist
- medical oncologist
- pathologist

1. Which of the following factors is affecting the patients´ prognosis the most?

- patients´ age
- patient´s co-morbidities
- biological behaviour of the tumour
- the tumour stage
- patient´s sex

1. Which of the following disciplines is NOT part of the general interdisciplinary / multimodal treatment approach in oncology:

- nephrology
- clinical oncology
- surgery
- pathology
- radiotherapy
- radiology

1. Which of the following statements about palliative care is NOT true?

- supports patients and their relatives
- palliative care maintains patient´s quality of life
- prolongs patient´s live expectancy
- palliative care focuses on symptom relief

1. The overall cancer mortality in a population can be significantly reduced by:

- more doctors
- cessation of smoking
- health promotion
- prostate cancer screening

1. What does neoadjuvant chemo- and/or radiotherapy mean?

- treatment of distant metastasis
- chemo- and/or radiotherapy after radical surgery
- radical surgery without any additional chemo- and/or radiotherapy
- chemo- and/or radiotherapy before radical surgery

SPECIFIC ASPECTS OF CANCER

1. Which chemotherapy is a typical alkylating agent?

- Cyclophosphamid
- Methotrexat
- Flououracil
- Vincristine

1. Which agent has the highest emetogenic potential?

- Gemcitabine
- Vincristine
- Erlotinib
- Cisplatin

1. Which side effect is typical for 5-FU?

- cholinergic syndrome
- skin rash
- increased transaminases
- hand and foot syndrome

1. What is the RBE in ion beam therapy?

- radioactive boson excitation
- radon body exposure
- relative biological effectiveness
- regulated beam energy

1. What is the Bragg peak?

- the perfect time point to start an ion beam treatment
- a boost to the residual treatment given by proton or carbon ion therapy
- a small mountain in Austria, not far from MedAustron
- the maximum of the characteristic energy loss curve of charged particles

1. What is NOT a technique used for radiotherapy treatment?

- Targeted Therapy
- Brachytherapy
- Proton Therapy
- Photon Therapy

1. Which one of the following cancers IS NOT an AIDS-defining cancer?

- Invasive cervical carcinoma
- Non-Hodgkin-Lymphoma (NHL)
- Kaposi Sarcoma
- Colorectal cancer

1. The cellular origin of the Kaposi's sarcoma (KS) is:

- Melanocytes
- T-lymphocytes
- Plasma cells
- venular endothelial cells

1. The following virus is detected in more than 90% of Primary CNS Lymphomas in HIV-infected patients:

- Epstein Bar Virus (EBV)
- Herpes simplex type 2 virus
- Human Papilloma Virus (HPV)
- Cytomegalovirus (CMV)

1. Which of the following is NOT a priority for patients with a life-limiting illness receiving palliative care?

- prolonging life at all costs
- strengthening relationships with loved ones
- relieving burden
- obtaining a sense of control

1. Which of the following is the most reliable indicator of pain?

- results of multidimensional assessment
- patients´ self-report
- results of functional assessment
- results of physical examination

1. When managing dyspnoea in patient with terminal disease

- providing supplemental oxygen should be the primary approach, even in patients without hypoxia
- non-pharmacological interventions are generally ineffective
- evidence supports the use of morphine for patients with advanced lung cancer
- the first step is the treatment of symptoms rather than the underlying cause

1. The following statement is correct for late complications of radiotherapy:

- their late time is depending on the radiation dose
- They are, in general, reversible
- They are exclusively based on radiation damage to the vasculature
- Their clinical manifestation is complete within the first year after radiotherapy

1. Radiosensitivity of organs and tissues:

- Tissues with a high proliferation rate are highly sensitive to changes in dose per fraction
- Changes in the overall treatment time of radiotherapy significantly impact on the severity of early complications
- The most radiosensitive normal tissue is the spinal cord
- The radiation sensitivity of tissues is closely related to their proliferation rate

1. The fractionation effect in normal organs and tissues:

- In all tissues, the *α/β* value remains constant throughout a series of fractionated irradiation
- The repair half-time of cells and tissues is comparable between cell cultures, rodent and human tissues
- The fractionation effect is the most pronounced for late responding tissues
- The fractionation effect is exclusively related to the DNA repair capacity of cells

1. Which percentage of all urothelial tumours presents in Upper Tract (Ureter, Kidney)?

- 10%
- 25%
- 15%
- 6%

1. Golden standard in management of high grade Upper Tract Urothelia Carcinoma located in Kidney Upper Calix is:

- Radiotherapy
- Nephroureterectomy and bladder cuff resection
- Nephrectomy
- Nephroureterectomy

1. Next step in patient with high suspicion of prostate cancer and negative transrectal biopsy is:

- Androgen deprivation therapy
- Radical prostatectomy
- re-biopsy
- PSA control in 3 months

1. What is the most common primary malignant intraocular tumour in adults?

- Uveal melanoma
- Metastatic lesions
- Retinoblastoma
- Intraocular lymphoma

1. What is the most common treatment for uveal melanoma?

- Evisceration of the affected eye
- radiotherapy
- Enucleation of the affected eye
- surgical resection of the tumour

1. What is the most common malignant ocular adnexal tumour?

- Basal cell carcinoma
- Orbital lymphoma
- Squamous cell carcinoma
- Conjunctival melanoma

1. Which is **not** a typical part of cancer rehabilitation?

- Radiation
- Psycho-Oncology
- Exercise
- Nutrition

1. Which is a typical part of cancer rehabilitation?

- Bioresonance
- Reiki
- Spiritual Healing
- Information

1. What kind of exercise is **not** typical for cancer rehabilitation?

- Strength exercise
- Senormotor training
- Weight management exercise
- Endurance exercise

1. Which of the following statements regarding targeted therapies is correct?

"Targeted therapy...

- ..takes advantage of genetic changes in malignant cells!"
- ...avoids normal cells and goes directly to the cancer cell!”
- …is mainly cytotoxic!"
- ...can not be combined with chemotherapy!"

1. Which of the following characteristics is NOT regarded as a hallmark of cancer?

- tumour promoting inflammation
- sustaining proliferative signals
- resistance against chemotherapy
- resisting cell death

1. Which statement about side effects in targeted therapies is correct?

- Severe side effects are never observed in targeted therapies!
- Can lead to interruption or stop of targeted therapy!
- Side effects only occur, if targeted therapy is combined with chemotherapy or radiation!
- Is mainly seen as skin toxicity!

1. Which epidemiologic fact about lung cancer is true?

- the lung cancer mortality is decreasing worldwide
- lung cancer is the leading cause of death in women in some countries
- only current smokers are affected by lung cancer
- lung cancer incidence is decreasing worldwide

1. Which screening method has the potential to reduce lung cancer mortality?

- sputum cytology
- chest x-ray
- low dose CT-scan
- measurement of exhale breath condensate

1. Which among the following risk factors shows the lowest influence on developing breast cancer?

- alcohol consumption
- high breast tissue density
- high dose radiation to the chest
- one first-degree relative with breast cancer

1. The most common first symptom of early breast cancer is:

- excretion of fluid from the nipple
- shortness of breast
- back pain
- painless mass or node in the breast

1. Median survival from diagnosis of metastatic breast cancer is

- 6 month
- 36 month
- 12 month
- 24 month

1. In locally advanced cervical cancer in which both parametriae are involved, the right one to the pelvic wall, the treatment of choice is:

- palliative treatment as the patient is too advanced to be cured
- neoadjuvant chemotherapy followed by radical hysterectomy
- definitive radio-chemotherapy
- radical hysterectomy and lymph node staging

1. Please select the true statement

- surgery is mandatory in all patients with gynaecological malignancies
- radiotherapy in the treatment of gynaecological malignancies could have a curative role in the treatment of endometrial cancer, cervical cancer and an adjuvant role in the treatment of ovarian cancer
- radiotherapy in the treatment of gynaecological malignancies is used only in rare cases in which surgery is contraindicated

1. Which of the following statements is NOT true:

- the main risk factors for developing uterine cancer are: obesity, DM II, hypertension, infertility
- vulva carcinoma is a very frequent malignancy in women
- the treatment of gynaecological malignancies requires a multidisciplinary approach
- ovarian cancer treatment requires extensive surgery followed by chemotherapy in the majority

1. Decision making in oncology is depending on.... Please indicate the wrong statement

- general condition of the patient
- histology
- amount and spared of gross tumour volume
- patient´s sex

1. Increase of secondary cancers occurs after ..... Please indicate the wrong answer

- chemotherapy
- chemo radiation
- radiotherapy
- surgery

1. What does LINAC stand for?

- Linear accelerator
- Long intense non-alcoholic cocktail
- Linear arc
- Tram line in Vienna
